# Head-to-head comparison of [^68^Ga]Ga-FAPI-04 and [^18^F]-FDG PET/CT in evaluating the extent of disease in gastric adenocarcinoma

**DOI:** 10.1007/s00259-021-05494-x

**Published:** 2021-07-24

**Authors:** Jonathan Kuten, Charles Levine, Ofer Shamni, Sharon Pelles, Ido Wolf, Guy Lahat, Eyal Mishani, Einat Even-Sapir

**Affiliations:** 1 Department of Nuclear Medicine, Tel Aviv Medical Center, affiliated to the Sackler School of Medicine, Tel Aviv University, 6 Weizmann St, 6423906 Tel Aviv, Israel; 2 Cyclotron Radiochemistry Unit, Hadassah Medical Organization and Faculty of Medicine, Hebrew University of Jerusalem, Jerusalem, Israel; 3 Division of Oncology, Tel Aviv Medical Center, affiliated to the Sackler School of Medicine, Tel Aviv University, Tel Aviv, Israel; 4 Department of Surgery, Tel Aviv Medical Center, affiliated to the Sackler School of Medicine, Tel Aviv University, Tel Aviv, Israel

**Keywords:** Gastric cancer, [^68^Ga]Ga-FAPI-04, [^18^F]-FDG, PET/CT, Peritoneal carcinomatosis, Head-to-head

## Abstract

**Background:**

[^18^F]-Fluorodeoxyglucose (FDG) positron emission tomography–computed tomography (PET/CT) may sometimes be suboptimal for imaging gastric adenocarcinoma. The recently introduced [^68^Ga]Ga-FAPI-04 (FAPI) PET/CT targets tumor stroma and has shown considerable potential in evaluating the extent of disease in a variety of tumors.

**Methods:**

We performed a head-to-head prospective comparison of FAPI and FDG PET/CT in the same group of 13 patients with gastric adenocarcinoma who presented for either initial staging (*n* = 10) or restaging (*n* = 3) of disease. Lesion detection and maximum standardized uptake value (SUV_max_) were compared between the two types of radiotracers.

**Results:**

All ten primary gastric tumors were FAPI-positive (100% detection rate), whereas only five were also FDG-positive (50%). SUV_max_ was not significantly different, but the tumor-to-background ratio was higher for FAPI (mean, median, and range of 4.5, 3.2, and 0.8–9.7 for FDG and 12.9, 11.9, and 2.2–23.9 for FAPI, *P* = 0.007). The level of detection of regional lymph node involvement was comparable. FAPI showed a superior detection rate for peritoneal carcinomatosis (100% vs. none). Two patients with widespread peritoneal carcinomatosis underwent a follow-up FAPI scan after chemotherapy: one showed partial remission and the other showed progressive disease.

**Conclusions:**

The findings of this pilot study suggest that FAPI PET/CT outperforms FDG PET/CT in detecting both primary gastric adenocarcinoma and peritoneal carcinomatosis from gastric cancer. FAPI PET/CT also shows promise for monitoring response to treatment in patients with peritoneal carcinomatosis from gastric cancer; however, larger trials are needed to validate these preliminary findings.

**Supplementary Information:**

The online version contains supplementary material available at 10.1007/s00259-021-05494-x.

## Introduction

Gastric cancer is the fifth most diagnosed cancer and the fourth leading cause of cancer death worldwide [1]. Prognosis largely depends upon achieving early diagnosis and accurate staging for offering the potential for complete resection of early-stage non-metastatic disease [2]. The role of [^18^F]-Fluorodeoxyglucose (FDG) positron emission tomography–computed tomography (PET/CT) in gastric cancer is unclear. FDG PET/CT has been shown to contribute clinically relevant information on nodal staging and metastatic status of the disease [2]. However, a wide range of sensitivity and specificity related to several factors has been reported, including physiological uptake in the stomach wall and uptake due to gastritis, as well as variable avidity of different histologic subtypes of gastric cancer to FDG, namely, diffuse-type, mucinous, and signet ring histology, which are notoriously known to have low-FDG avidity. These, in turn, are attributed to mechanisms, such as relative acellularity and low expression of glucose transporter 1 protein [3]. FDG PET/CT has also been shown to be limited in evaluating the extent of disease in the peritoneal carcinomatosis pattern of metastatic disease that is frequently encountered in gastric cancer [4]. As in other malignancies, false-negative FDG PET results can also be observed in hyperglycemic patients or in subcentimeter lesions.

Stromal cells, which are composed mainly of cancer-associated fibroblasts (CAFs), contribute up to 90% of the gross tumor mass. CAFs differ from normal fibroblasts by expressing proteins not found in their normal counterparts, including fibroblast activation protein (FAP). This gives them pro-tumorigenic activity, such as enabling invasion and migration of tumor cells, involvement in tumor angiogenesis, activation of cell signaling, decreasing of the chemotherapeutic drug uptake, and bestowing an immunosuppressive role. FAP is expressed in more than 90% of human epithelial cancers and is nearly absent from normal tissues in adult humans [5, 6].

Based on these characteristics of the tumor stroma, new radiotracers consisting of FAP-specific small molecule inhibitors (FAPI) have been developed for both tumor imaging and therapy, and their application has been described for imaging of various malignancies, including those that are not FDG-avid, and for showing a significantly high tumor-to-background contrast [4, 7–12].

The aim of the present small prospective study was to compare the findings of FDG with those of FAPI PET/CTs in the same patients with gastric adenocarcinoma.

## Material and methods

### Patients

Between July and December 2020, patients with gastric cancer referred for either staging or restaging of disease were prospectively and consecutively recruited and underwent both FDG PET/CT and FAPI PET/CT scans. The patients were enrolled as part of a larger ongoing study in our institution to evaluate the role of FAPI PET/CT in the imaging of various malignancies (ClinicalTrials.gov identifier: NCT04441606). Two of the patients had also undergone a second FAPI PET/CT scan, to evaluate response to treatment, and those results were analyzed as well. The study was approved by the institutional review board (TLV-0308–20), and all patients gave written informed consent to participate in the trial.

Fifteen patients were enrolled into the study. Two patients were excluded from the final analysis due to the absence of a definitive diagnosis of gastric adenocarcinoma. One of them had actually undergone a revision of the initial pathology report due to negative FAPI PET/CT and FDG PET/CT scans which failed to detect any malignancy. The other patient had an FDG- and FAPI-positive gastric mass; however, his biopsy was inconclusive and the patient refused further workup or treatment. Thirteen patients were available for analysis (six men and seven women; median age 70 years, range 35–87), all with gastric adenocarcinoma (proven by gastroscopy guided biopsies) and four with signet ring morphology (31%).

Ten (77%) treatment-naïve patients were referred for initial staging, and three (23%) patients were referred for restaging prior to decision on further management (the latter three had undergone a gastrectomy for removal of the primary tumor with or without metastasectomy and chemotherapy). The FAPI PET/CT scans were tolerated well by all patients, and no adverse events were encountered during or after [^68^Ga]Ga-FAPI-04 injection.

### *[*^*68*^*Ga]Ga-FAPI-04 synthesis*

[^68^Ga]Ga-FAPI-04 was prepared for research purposes according to a fully validated process that used the iTM ^68^Ge/^68^ Ga generator and iQS-TS automated synthesis module (Isotope Technologies Munich GmBH, Munich, Germany), as previously described with minor modifications [5, 13]. A full description of the synthesis process is available in the supplemental material.

### Imaging protocol

All of the patients were scanned with the same Discovery MI PET/CT system (GE Healthcare) scanner. The median time between the FDG and the FAPI scans was 6 days (range 1–23 days). The injected doses of each radiotracer were weight-adjusted (3.7 MBq (0.1 mCi)/kg for FDG and 1.8–2.2 MBq (0.05–0.06 mCi)/kg for FAPI-04). The patients were instructed to fast for at least 4 h and to avoid physical exercise for 24h prior to FDG administration, and glucose was tested to ensure a normal blood glucose level (< 150 mg/dl). The PET/CT scans were acquired 1 h after intravenous injection of the radiotracer. Proper hydration was ensured prior to all scanning procedures, and the patients were instructed to void immediately prior to imaging acquisition.

The CT scans were performed with automatic mA-modulation and 120 kV and reconstructed to a slice thickness of 2.5 mm. Contrast material was administered orally and intravenously in at least one of the scans. PET acquisition was performed with an acquisition time of 4 min per bed position for FAPI and 3 min per bed position for FDG. The scans were reconstructed in a matrix size of 256 × 256 with a pixel size of 2.7 mm and a slice thickness of 2.8 mm. The reconstruction method used time of flight information and included normalization and image corrections for attenuation, scatter, randoms, and dead time. The data were reconstructed by means of the Bayesian penalized likelihood reconstruction algorithm (Q.clear; GE Healthcare), with a penalization factor (β)of 500 for FDG and 750 for FAPI.

### Image analysis

All of the scans were interpreted jointly in consensus by a nuclear medicine physician (EES) and a body radiologist (CL), each with decades of experience in reading oncologic PET/CT images. The studies were read with the Xeleris workstation (GE Healthcare), which allows the review of PET, CT, and fused imaging data in axial, coronal, and sagittal slices.

Pathologic uptake in the primary stomach tumor was considered a focal uptake higher than that of the background stomach activity. Pathological uptake in involved lymph nodes and other metastatic lesions was judged as uptake exceeding the activity of its adjacent background tissues. Anatomical data from each CT scan were accounted for in the interpretation of all lesions. The maximum standardized uptake value (SUV_max_) in malignant lesions was automatically calculated by means of a 1 cm^3^ spherical volume of interest. For the primary gastric tumor, uptake in a similar volume of interest in a non-involved area of the stomach was used as background activity to calculate the tumor-to-background ratio. The average SUV_max_ of the five lesions with the most intense uptake was calculated in patients with peritoneal carcinomatosis.

### Reference standard

Clinical follow-up, surgical macroscopic and histological findings, and follow-up imaging examinations were considered reference standard. Features on follow-up imaging studies that were considered validation of the malignant nature of lesions were either progression of metastatic disease or response to chemotherapy in terms of reduction in size and/or number of lesions.

### Statistical analysis

Continuous variables were evaluated for normal distribution with histograms and Q-Q plots. Categorical variables were reported as frequency and percentage, and continuous variables were reported as mean, median, and range. The Wilcoxon signed-rank test was applied to compare SUV_max_ values between the FAPI and FDG scans. The test was two-sided, and *P* < 0.05 was considered statistically significant. SPSS was used for all statistical analyses (IBM Corp. Released 2020. IBM SPSS Statistics for Windows, version 27.0. Armonk, NY: IBM Corp).

## Results

### Initial staging

All 10 patients referred for initial staging had a FAPI-positive primary gastric tumor (100%), but only 5 (50%) were also interpreted as FDG-positive, while the others had faint or no FDG uptake in their primary tumor. The tumor-to-background ratio was significantly higher for FAPI. The mean, median, and range of SUV_max_ for the primary tumors were 11.6, 5.5, and 1.6–32 for FDG, and 16.6, 15.9, and 4–32 for FAPI (*P* = 0.139). The mean, median, and range of the tumor-to-background ratio were 4.5, 3.2, and 0.8–9.7 for FDG and 12.9, 11.9, and 2.2–23.9 for FAPI (*P* = 0.007). None of the patients who had an FDG-negative primary tumor had FDG-positive lymph nodes or metastases.

**Fig. 1:**
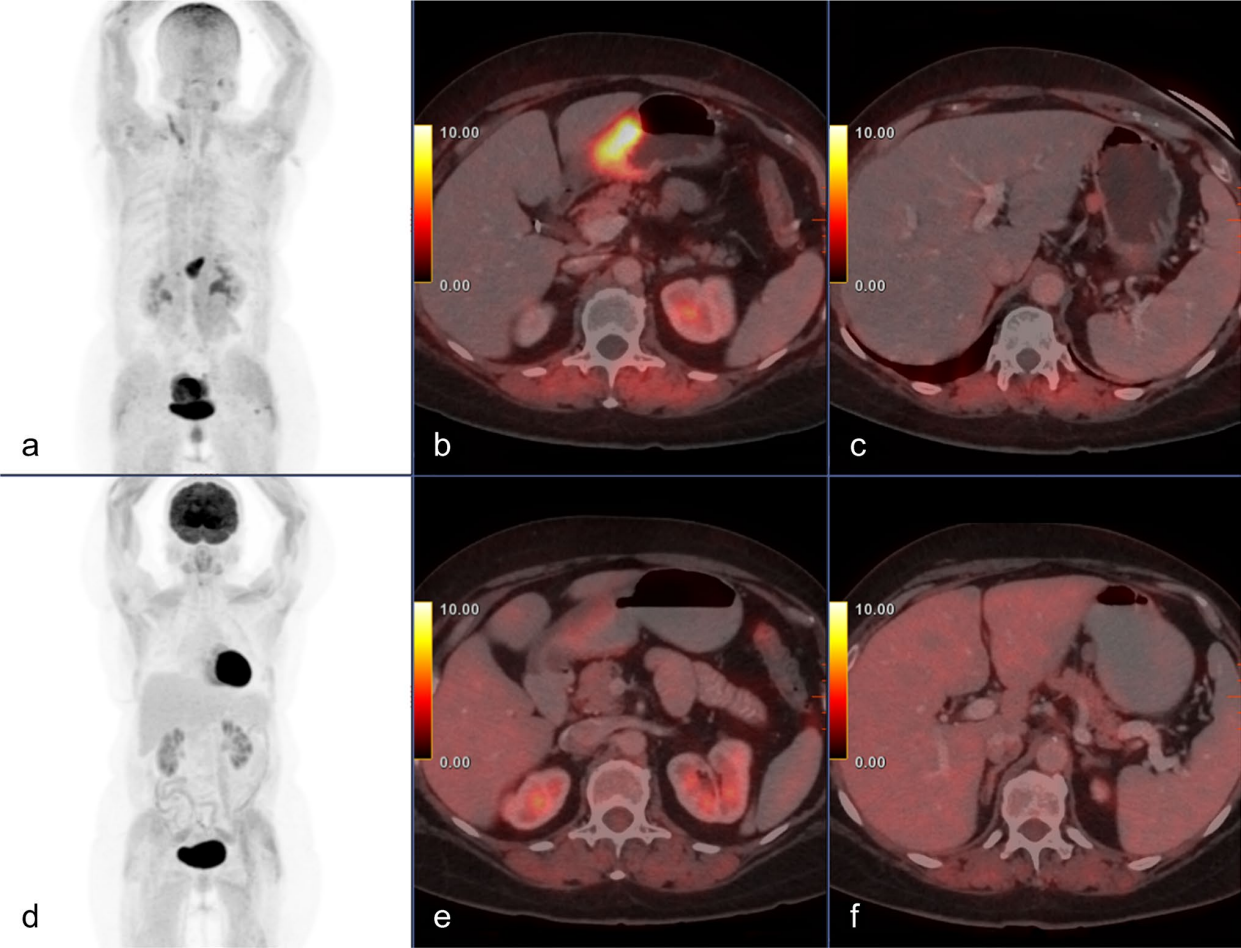
Staging FAPI (**a**,** b**,** c**) and FDG (**d**,** e**,** f**) PET/CT scans of a 65-year-old female with poorly differentiated gastric adenocarcinoma (patient #8 Table 1), showing a FAPI-positive, FDG-negative primary tumor (SUV_max_ 11.8 and 2.3, respectively) and a perigastric lymph node (SUV_max_ 0.3 and 2.3, respectively). She was treated with neoadjuvant chemotherapy and had stable disease on follow-up imaging

Patient characteristics and imaging findings for the staging group are summarized in Table 1. Figure 1 shows an example of a patient with a FAPI-positive and an FDG-negative tumor on staging. Both FAPI PET/CT and FDG PET/CT detected the same 16 positive regional lymph node metastases in two patients. FAPI PET/CT detected an additional positive lymph node in one patient whose primary tumor was not FDG-avid. The mean, median, and range of SUV_max_ in metastatic lymph nodes were 4.9, 4.3, and 0.3–10.1 for FDG and 6.5, 7.9, and 2.3–9.2 for FAPI (*P* = 0.285).

**Table 1:** Patient characteristics and imaging findings of the staging group (*n* = 10)

n	Age (y)	Histology	Primary tumor			LN num. mean SUV_max_		PC mean SUV_max_		Follow-up	CA-125 (U/ml)	CA-19–9 (U/ml)	CEA (ng/ml)
				FDG	FAPI	FDG	FAPI	FDG	FAPI				
1	79	Mod. diff	SUV_max_	32	22	0	0	n/a	n/a	Surgery 0/14 LN	2.9	8.6	< 1.3
			T:B	9.7	12.9								
2	78	Poorly diff	SUV_max_	6.8	23	0	0	2.3	7.5	Chemotherapy FAPI PET/CT—PD	31	3.7	2.8
			T:B	3.8	11.5								
3	87	Unknown diff	SUV_max_	21	32	0	0	n/a	n/a	Radiotherapy FDG PET/CT—PR	35.3	50.9	23.2
			T:B	7.8	17.8								
4	59	Mod. diff. signet ring	SUV_max_	3.9	16.7	0	0	n/a	n/a	Surgery 14/56 LN CT—PD	8.6	6.5	< 1.3
			T:B	2.6	23.9								
5	72	Poorly diff	SUV_max_	4	22	0	0	1.6	13.9	Dead of disease	n/a	74	1.4
			T:B	1.8	22								
6	70	Poorly diff	SUV_max_	12.8	14.5	12 (4.3)	12 (7.9)	n/a	n/a	Na chemo FDG PET/CT—PR Surgery 11/16 LN	15.6	440	16.2
			T:B	7.1	12								
7	73	Well diff	SUV_max_	4.2	4	0	0	n/a	n/a	Surgery 0/11 LN	6.4	60	3.4
			T:B	1.2	2.2								
8	65	Poorly diff. diffuse type	SUV_max_	2.3	11.8	0 (0.3)	1 (2.3)	n/a	n/a	Chemotherapy FDG PET/CT- SD	n/a	n/a	n/a
			T:B	1.2	11.8								
9	66	Unknown diff	SUV_max_	1.6	5.5	0	0	n/a	n/a	Na chemo	n/a	n/a	n/a
			T:B	0.8	5.5								
10	42	Poorly diff	SUV_max_	27	15.2	4 (10.1)	4 (9.2)	n/a	n/a	Na chemo	13	17	< 1.3
			T:B	9	8.9								

*Mod* moderately, *diff.* differentiated, *LN* lymph nodes, *PC* peritoneal carcinomatosis, *NA* neoadjuvant, *PD* progressive disease, *PR* partial remission, *SD* stable disease, *T:B* tumor-to-background ratio; tumor marker reference values: CA-125: 0–35; CA-19–9: 0–32; CEA: 0–5

Both patients with FAPI-avid and FDG-avid lymph nodes underwent exploratory laparotomies to rule out distant peritoneal metastases, and both were treated with chemotherapy. One of them also had a follow-up FDG PET/CT scan which showed reduced size and uptake of all involved lymph nodes, and later underwent subtotal gastrectomy and lymph node dissection that yielded 11 positive regional lymph nodes out of a total of 16. The patient with one FAPI-positive and one FDG-negative lymph node was treated with neoadjuvant chemotherapy and had an FDG PET/CT follow-up scan that showed stable FDG-non-avid disease. Five patients had neither lymph node nor peritoneal involvement in either imaging study. Three of them underwent neoadjuvant chemotherapy and gastrectomy: two patients had no positive lymph nodes per pathology (0/25), and one patient with a FAPI-positive and an FDG-negative primary tumor had 14/56 positive perigastric lymph nodes which were considered being false-negatives probably due to microscopic spread. Two patients had peritoneal carcinomatosis on their staging FAPI scans. One was treated with neoadjuvant chemotherapy and underwent a follow-up FAPI PET/CT scan, and the other one was followed for 3 months, during which she deteriorated clinically until she passed away.

Images of the histopathological analysis of the primary tumor of two patients (pt. 1 and pt. 4 in the staging group) are available as supplemental Fig. 1.

### Restaging

All three patients referred for restaging had poorly differentiated signet ring gastric adenocarcinoma. They all showed peritoneal carcinomatosis with diffuse FAPI uptake and negative FDG scans that demonstrated minimal or no uptake aside from some non-specific uptake in areas of the bowel wall. They were treated with chemotherapy, and each had a follow-up imaging examination that confirmed the results of the previous FAPI PET/CT scan; i.e., two patients showed progression on CT or on the CT part of an FDG (non-avid disease) PET/CT, and one patient underwent a follow-up FAPI PET/CT scan 5 months after the first scan and it showed partial remission. The latter also underwent an exploratory laparotomy after the first scan that had found a peritoneal cancer index of 30, confirming the FAPI PET/CT results. The patient characteristics and imaging findings for the restaging group are summarized in Table 2.

**Table 2:** Patient characteristics and imaging findings of restaging (*n* = 3)

Patient	Age (y)	Histology	Peritoneal carcinomatosis mean SUV_max_		Follow-up	CA-125 (U/ml)	CA-19–9 (U/ml)	CEA (ng/ml)
			FDG	FAPI				
1	35	Poorly diff. signet ring	2.5	8.8	CT–PD	19.5	4.3	< 1.3
2	71	Poorly diff. signet ring	3	10	FDG PET/CT–PD	118.5	39.2	< 1.3
3	57	Poorly diff. signet ring	2.8	9.6	FAPI PET/CT–PR	6.5	34.8	2.7

*diff* differentiated, *PD* progressive disease, *PR* partial remission; tumor marker reference values: CA-125: 0–35; CA-19–9: 0–32; CEA: 0–5

### Peritoneal carcinomatosis

The five patients (38.5%) who had peritoneal carcinomatosis included the three patients who presented for restaging and two of the patients who presented for initial staging, as described above. All five had poorly differentiated tumors, and three had signet ring morphology. They all showed diffuse FAPI uptake in the peritoneal cavity, with minimal or no FDG uptake aside from some non-specific uptake in areas of the bowel wall. The mean, median, and range of SUV_max_ in the peritoneal carcinomatosis lesions were 2.4, 2.5, and 1.6–3 for FDG and 10, 9.6, and 7.5–13.9 for FAPI (*P* = 0.043).

### Monitoring response to chemotherapy

**Fig. 2:**
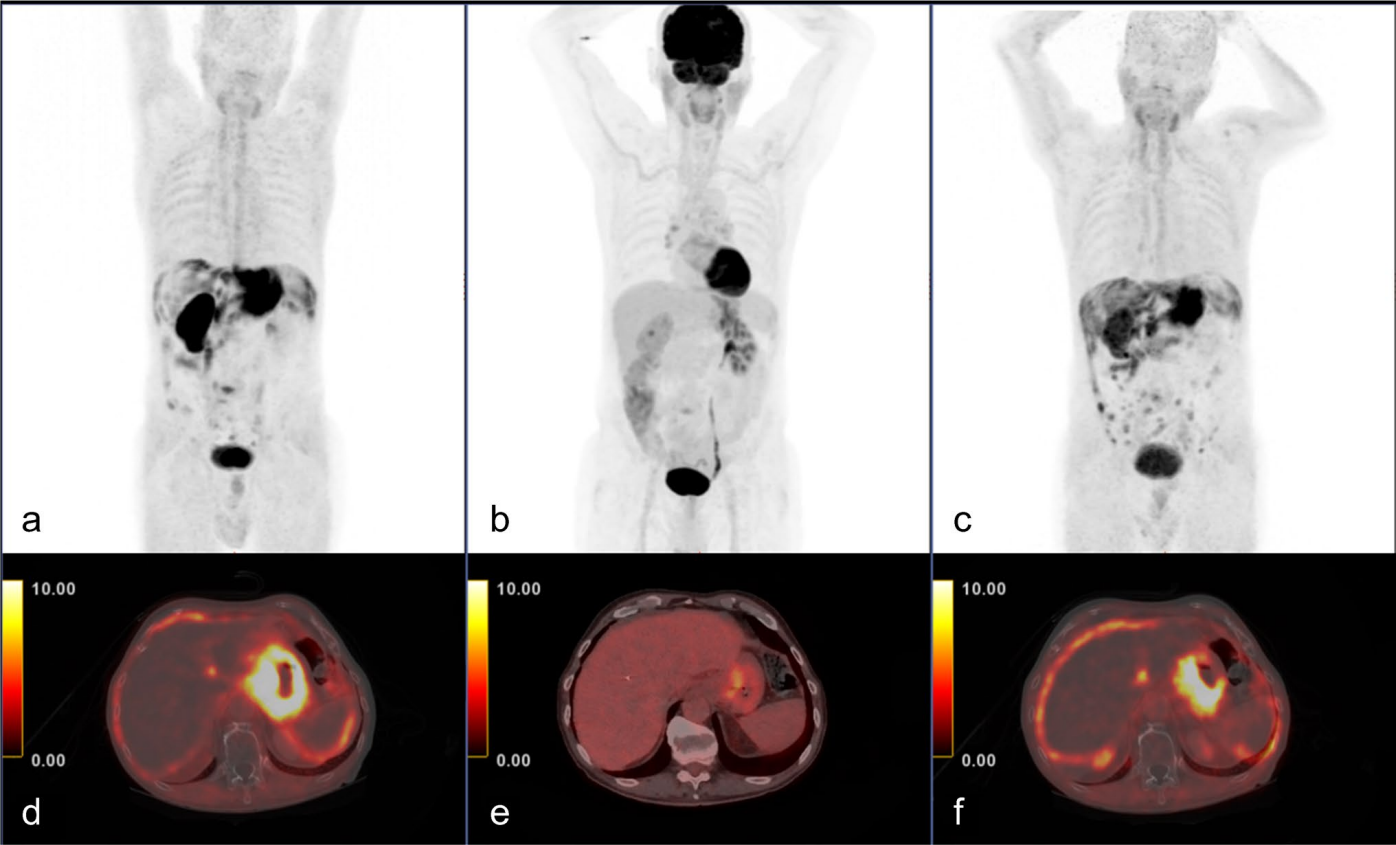
Staging FAPI (**a**,** d**) and FDG (**b**,** e**) PET/CT scans of a 78-year-old male with poorly differentiated gastric adenocarcinoma (patient #2 Table 1) showing intense FAPI uptake in the primary tumor and in peritoneal carcinomatosis compared with FDG (primary tumor SUV_max_ 23 and 6.8; primary tumor tumor-to-background ratio; 11.5 and 3.8; peritoneal-carcinomatosis SUV_max_ 7.5 and 2.3, respectively). The follow-up FAPI PET/CT (**c**,** f**) after 4 months of chemotherapy shows disease progression

Two patients with peritoneal carcinomatosis underwent a follow-up FAPI PET/CT scan after neoadjuvant chemotherapy. One patient showed disease progression manifested as new implants and more diffuse peritoneal fat infiltration after 4 months of chemotherapy, confirming the true positive results of the initial FAPI PET/CT scan. The average SUV_max_ increased slightly, from 7.5 to 8 (Fig. 2). The other patient had partial remission after 5 months of treatment, with most lesions having been undetected and only mild FAPI uptake identified in a few residual implants. That patient’s average SUV_max_ decreased from 9.6 to 2.3.

## Discussion

The recently introduced FAP-targeting PET radiotracers are emerging as potential alternatives to the well-established FDG PET/CT for evaluating a variety of malignancies, especially those in which FDG is known to be of limited value [4, 7–11, 14]. In the present prospective study, FAPI and FDG PET/CTs were compared head-to-head in the same patients with gastric adenocarcinoma, a common malignancy known to show variable FDG avidity. In this small cohort, FAPI outperformed FDG in detecting the primary gastric tumor, showing a 100% detection rate as opposed to only 50% for FDG. It was also superior in detecting the presence and extent of peritoneal carcinomatosis, with a 100% detection rate vs. none. The rate of the detection of regional lymph node involvement was comparable for the two types of radiotracers. FAPI showed higher uptake than FDG for peritoneal lesions as well as a higher tumor-to-background uptake ratio for primary gastric tumors due to the minimal background uptake of FAPI in the abdominal viscera.

Our results are in line with those of Pang et al. [11] who recently showed FAPI PET/CT to be superior to FDG PET/CT in detecting primary and malignant lesions in a heterogeneous group of patients, including several with gastric cancer. The high detection rate shown here for FAPI in primary gastric tumors of variable differentiation levels together with the known limitations of FDG in detecting several subtypes of gastric carcinoma, such as mucinous, poorly differentiated, and signet ring tumors [3], raises the possibility of using FAPI as a preferred radiotracer for evaluating gastric cancer. However, due to the small size of the present cohort, this possibility needs to be further explored in larger trials before any conclusion could be reached. The significantly superior tumor-to-background ratio uptake in primary gastric tumors shows promise for the use of FAPI PET/CT for the delineation of tumors for radiotherapy, as has already been shown in other types of tumors [8, 15].

In a small subgroup analysis, FAPI outperformed FDG in detecting and evaluating the extent of poorly differentiated signet ring gastric adenocarcinoma with peritoneal carcinomatosis. FDG showed especially poor performance in that subgroup, largely due to its relatively high physiological background uptake in the bowel wall and other abdominal viscera. In contrast, FAPI was remarkably useful in that subgroup, with high uptake in diffuse peritoneal metastatic spread and minimal-to-no background activity. This superior sensitivity of FAPI over FDG in detecting peritoneal carcinomatosis was recently reported by Zhao et al. [4] in a cohort of patients with different types of cancers.

In the present study, two patients with widespread peritoneal carcinomatosis who were FAPI-positive and FDG-negative had undergone a second follow-up FAPI PET/CT scan after several months of chemotherapy. One of them had partial remission, while the other had disease progression. Although only anecdotal, this points to some potential for applying FAPI PET/CT to monitor response to treatment, specifically in peritoneal carcinomatosis, a possibility that has hardly been explored in depth to date [16].

There are several limitations to the present trial, the first of which is the small number of patients included in the cohort (*n* = 13). Second, patients presenting for initial staging as well as those with widespread peritoneal metastatic disease presenting for restaging were included. Third, none of the patients in the current trial had distant metastatic disease (i.e., outside of the peritoneal cavity) or any hepatic involvement; thus, no conclusion could be drawn on the detection of those lesions.

In conclusion, the results of the present pilot study suggest that FAPI PET/CT outperforms FDG PET/CT in detecting primary gastric adenocarcinoma, as well as in detecting peritoneal carcinomatosis with a gastric cancer origin. FAPI PET/CT has also shown promise in monitoring response to treatment in patients with peritoneal carcinomatosis; however, larger trials are needed to validate these preliminary findings.

## Supplementary Information

Below is the link to the electronic supplementary material.

Supplementary file1 (DOCX 24 KB)

Supplementary file2 (DOCX 2158 KB)

## Data Availability

The datasets used and/or analyzed during the current study are available from the corresponding author upon reasonable request.

## Abbreviations

PET/CT: Positron emission tomography–computed tomography
CT: Computed tomography
CAFs: Cancer-associated fibroblasts
FAP: Fibroblast activation protein
SUV_max_: Maximum standardized uptake value
