# Supplementary material for: Head-to-head comparison of [68Ga]Ga-FAPI-04 and [18F]-FDG PET/CT in evaluating the extent of disease in gastric adenocarcinoma
Source: Eur J Nucl Med Mol Imaging. 2021 Jul 24;49(2):743–50. doi: 10.1007/s00259-021-05494-x (PMC8803763; doi:10.1007/s00259-021-05494-x)
Supplement: Supplementary file 1 — Supplementary file1 (DOCX 24 KB) [file 259_2021_5494_MOESM1_ESM.docx]

**[^68^Ga]Ga-FAPI-04 Synthesis**

[^68^Ga]Ga-FAPI-04 was prepared for research purposes in a fully validated process, using the iTM ^68^Ge/^68^Ga generator and iQS-TS automated synthesis module (Isotope Technologies Munich GmBH, Munich, Germany), as previously described with minor modifications[^1^](#_ENREF_1)^,^ [^2^](#_ENREF_2). In brief, ^68^Ga^3+^ (half-life 68 min; β+ 89%; Eβ+ max. 1.9 MeV) was eluted from the ^68^Ge/^68^Ga radionuclide generator (1850 GBq), to the reaction vessel, using 5 mL of hydrochloric acid (0.05 M). The preheated reaction vessel, contained 50 μg of DOTA-FAPI-04 precursor (Huayi isotopes Company, Jiangsu, China), dissolved in 1 mL of sodium acetate buffer (0.25 M). Radiolabeling was performed by 5 minutes incubation at 95 °C, using a disposable cassette and labeling kit (iTM GmBH, Munich, Germany). The reaction mixture was then loaded onto a SPE Sep-Pak C18 cartridge (pre-activated using 1.5 mL of ethanol solution, followed by 4 mL of 0.9 % sodium chloride solution). The SPE cartridge was subsequently washed with 5 mL of 0.9 % sodium chloride solution. [^68^Ga]Ga-FAPI-04 was eluted using 2 mL of 50 % (v/v) ethanol in ultra-pure water, and further diluted to a total final volume of 15 mL with 0.9 % sodium chloride solution. The final product was filter sterilized using a 0.22 μm syringe filter (Cathivex-GV, Darmstadt, Germany) and an aliquot of the final product solution was subjected to quality control. The final [^68^Ga]Ga-FAPI-04 product was a clear and colorless solution, with a pH of 4.0-8.0, and an activity of 1.092 ± 0.17 GBq (n=25; at the end of synthesis). Radiochemical identity & purity and chemical purity were assessed using a Shimadzu analytical HPLC system (Model LC20AD, Shimadzu, Kyoto, Japan). The system was equipped with C18 column (5 μm, 10 mm × 250 mm, Luna, Phenomenex, Torrance, CA, USA), UV detector operating at 220 nm, and a radio-detector (Bioscan B-FC 3200, Eckert & Ziegler Radiopharma, MA, USA). An eluent mixture of 0.1% (v/v) trifluoroacetic acid (TFA)/HPLC water (A) and 0.1 % (v/v) TFA/acetonitrile (B) was used in the following gradient; (t=0) 5% B, (t=14) 45 % B, (t=15) 45 % B, (t=17) 5 % B, (t=19) 5 % B. The flow rate was set to 1 mL/min and the column temperature to 25 °C. The [^68^Ga]Ga-FAPI-04 identity (retention time 8.8 min) was confirmed, using a non-radiolabeled reference standard, Ga-FAPI-04 (Huayi isotopes Company, Jiangsu, China; retention time 8.6 min). The radiochemical purity was 99.9 ± 0.15 % and the free ^68^Ga^3+^ content (retention time 1.47 min) in the final product, was 0.04 ± 0.15 %. A 48 hours retention sample revealed that the [^68^Ge] percentage was less than 0.001 % of the total activity content in the original sample. The radionuclidic identity was confirmed using half-life measurements and gamma spectrum analysis of the main product peak. Sterility and bioburden were assessed using, filter integrity tests, bacterial endotoxins tests, and microbial growth tests. The ethanol content in the final product was < 10 %, as verified by gas chromatography during separate process validations.

1. Lindner, T., et al., *Development of Quinoline-Based Theranostic Ligands for the Targeting of Fibroblast Activation Protein.* J Nucl Med, 2018. **59**(9): p. 1415-1422.

2. Loktev, A., et al., *A Tumor-Imaging Method Targeting Cancer-Associated Fibroblasts.* J Nucl Med, 2018. **59**(9): p. 1423-1429.
