# Supplementary material for: Head-to-head comparison of [68Ga]Ga-FAPI-04 and [18F]-FDG PET/CT in evaluating the extent of disease in gastric adenocarcinoma
Source: Eur J Nucl Med Mol Imaging. 2021 Jul 24;49(2):743–50. doi: 10.1007/s00259-021-05494-x (PMC8803763; doi:10.1007/s00259-021-05494-x)

Supplemental Fig1.

Histological analysis of the primary gastric tumor from two patients who underwent surgery (patients 1 and 4). Both case 1 (Figure a and b, X20 and X200 magnification, respectively) and case 2 (Figure c and d, X20 and X200 magnification, respectively) shows highly infiltrative tumors with involvement of the muscularis propria and significant desmoplastic reaction.


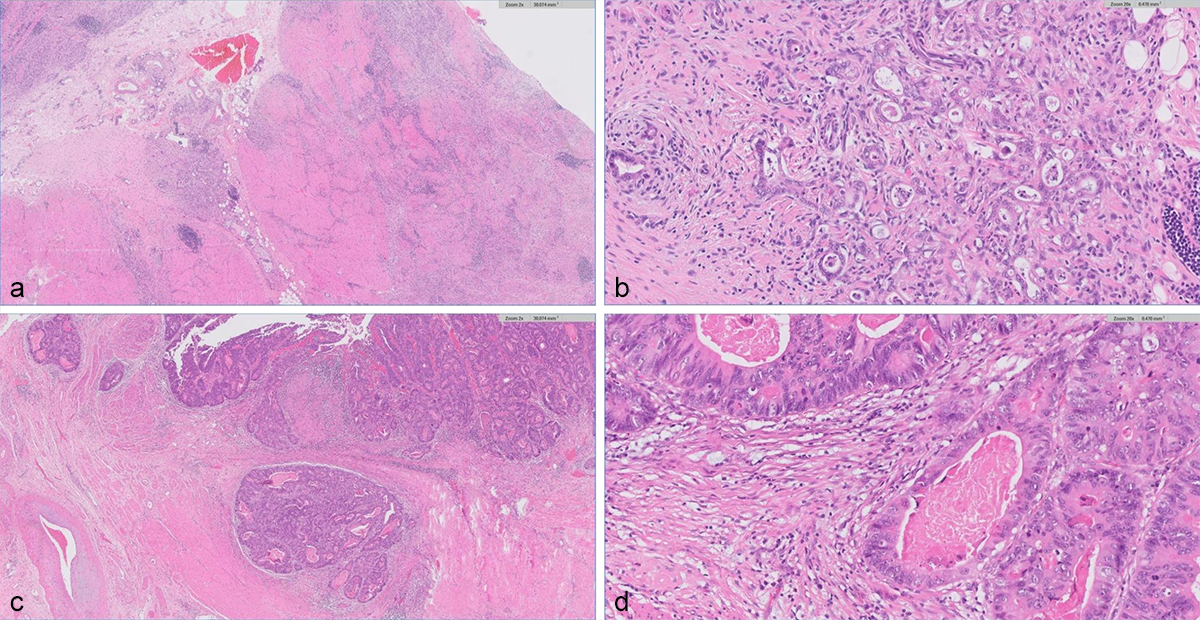

Supplement: Supplementary file 2 — Supplementary file2 (DOCX 2158 KB) [file 259_2021_5494_MOESM2_ESM.docx]
